# CANCER AND AGING RESEARCH FROM DIAGNOSIS TO SURVIVORSHIP

**DOI:** 10.1093/geroni/igae098.0991

**Published:** 2024-12-31

**Authors:** Sean Halpin

**Affiliations:** Chair

## Abstract

The patient’s cancer journey is a complex and multifaceted involving many emotional, environmental, and structural challenges—all of which are further complicated by advanced age. In our symposium, we bring together researchers who apply quantitative and qualitative methods to studying multiple cancer types from diagnosis to survivorship. First, Halpin will discuss the use of discursive psychology to examine how patient medical education is delivered to patients with multiple myeloma in preparation for autologous stem cell transplant. Second, Carrion will present on the treatment experience of immigrant Latinas and cancer. Next, Chidebe will apply interpretive phenomenological analysis to examine the impact of belonging to a peer support group for older Nigerian women living with metastatic breast cancer. Last, Krok-Schoen will present using cross-sectional data to examine age differences in severity and correlates of symptoms and quality of life among older breast and colorectal cancer survivors. Examining how a variety of methodological approaches are applied to explore the patient’s cancer journey will help facilitate a broader understanding of gerontological cancer research. Cancer and Aging Interest Group Sponsored Symposium

